# Designing a Capacitive Sensor to Detect Series Arcs in Aircraft HVDC Electrical Systems

**DOI:** 10.3390/s25164886

**Published:** 2025-08-08

**Authors:** Gema Salinero, Guillermo Robles

**Affiliations:** Department of Electrical Engineering, University Carlos III of Madrid, 28911 Leganés, Spain; gsaliner@ing.uc3m.es

**Keywords:** capacitive sensor, sensor design, capacitive coupling, series arc, aircraft, power systems

## Abstract

The transition toward more electric aircraft (MEA) and all-electric aircraft (AEA) has driven the adoption of high-voltage DC (HVDC) electrical architectures to meet increasing power demands while reducing weight and enhancing overall efficiency. However, HVDC systems introduce new challenges, particularly concerning insulation reliability and the detection of in-flight series arc faults. This paper presents the design and evaluation of a capacitive sensor specifically developed to detect series arc faults in HVDC electrical systems for aerospace applications. A model of the sensor is proposed and validated through both simulations and experimental measurements using a step response test. The results show excellent agreement between the model and the physical setup. After validating the capacitive coupling value and its response to high-frequency signals, series arcs were generated in the laboratory to evaluate the sensor’s performance under realistic operating conditions, which involve different signal dynamics. The results are highly satisfactory and confirm the feasibility of using capacitive sensing for early arc detection, particularly aligned with the stringent requirements of more electric aircraft (MEA) and all-electric aircraft (AEA). The proposed sensor thus enables non-intrusive detection of series arc faults in compact, lightweight, and safety-critical environments.

## 1. Introduction

In recent decades, the aerospace industry has undergone a structural transformation toward the more electric aircraft (MEA) concept. This shift is driven by two main imperatives: the growing environmental pressure to reduce greenhouse gas emissions from air transport and the economic need to improve operational efficiency and lower fuel and maintenance costs [1].

Environmental concerns have played a pivotal role in accelerating this transition. The MEA approach emerged in part as a response to advocacy from environmental organizations and regulatory bodies promoting aviation decarbonization, supported by governmental subsidies and financial incentives [1] such as those from the EU’s Clean Sky and FAA’s CLEEN programs [2]. Technologies such as electric taxiing systems and the replacement of traditional pneumatic and hydraulic subsystems with electrical alternatives have shown measurable impacts in reducing emissions and noise, particularly during low-speed ground operations [1,3].

From an economic perspective, moving toward more electric architectures leads to significant reductions in aircraft structural weight by eliminating complex hydraulic components and replacing them with distributed electrical systems [1]. For instance, MEA implementations can yield fuel savings in the range of 4–8% for environmental control system electrification, as demonstrated in Airbus A320 case studies [4]. This results in lower fuel consumption, increased payload capacity, and enhanced operational reliability [1]. Additionally, electric systems simplify maintenance procedures, reduce the number of potential failure points, and lower the overall operational costs throughout the aircraft’s life cycle.

The electrification of aircraft systems has undergone a progressive transformation from conventional architectures based on mechanical, hydraulic, and pneumatic systems to increasingly electric configurations. Traditional aircraft relied primarily on low-voltage DC systems (28 V DC) with limited power capacity, often supported by commutator-based generators. These systems were bulky, inefficient, and imposed high maintenance demands. As aviation technology advanced, aircraft such as the F-16 adopted three-phase AC architectures (115 V, 400 Hz), improving onboard power availability and system reliability [5]. More recent platforms transitioned to high-voltage DC (HVDC) systems (e.g., 270 V), significantly reducing wiring weight and enabling the replacement of hydraulic systems with electric alternatives, thus enhancing energy efficiency and reliability [1]. This transition laid the foundation for the more electric aircraft (MEA) concept, wherein increasing portions of onboard systems, i.e., flight control surfaces, landing gear, braking systems, are electrically driven. As a further step, aircraft propulsion architectures have diversified into turbo-electric, hybrid-electric, and all-electric designs. Turbo-electric systems use gas turbines to drive electrical generators feeding HVDC grids that power propulsion motors. Hybrid-electric systems combine conventional engines with electrical machines, using batteries or fuel cells to assist or fully drive propulsion, depending on the configuration (parallel, series, or series–parallel). In all-electric architectures, all propulsion power is supplied exclusively by electrical energy storage, typically batteries or hydrogen fuel cells, with energy managed via advanced DC/DC converters and motor inverters. These configurations vary in voltage levels, network centralization, and the integration of distributed propulsion systems, reflecting a continuous effort to optimize weight, efficiency, and reliability under aviation-specific constraints [1,3].

The transition toward MEA has been made possible through several modern platforms in both civil and military aviation. A leading example is the Boeing 787 Dreamliner, which replaced traditional pneumatic systems with electrically driven compressors and employs high-voltage variable-frequency electrical generators to power critical subsystems. With an installed electric power capacity exceeding 1 MW, it is considered the first large-scale commercial MEA, significantly reducing weight and maintenance requirements through decentralized power distribution architectures [1,3]. Similarly, the Airbus A350 and A380 families incorporate multiple AC and DC power buses (e.g., ±270 V DC and 115/230 V AC) [6], integrating electric actuation and environmental systems, although still retaining some pneumatic components [1,7]. In the military sector, aircraft such as the F-22 Raptor and upgraded helicopters like the Mi-24 adopted high-voltage DC architectures (e.g., 270 V DC), replacing hydraulic actuators with electromechanical alternatives and improving redundancy and fault tolerance via distributed electric power generation systems [1,3]. Emerging all-electric (AEA) and hybrid-electric aircraft [8,9] include the Pipistrel Velis Electro (EASA-certified), Diamond eDA40, and Bye Aerospace eFlyer 800, which demonstrate feasibility in training and regional markets. Meanwhile, conceptual projects like Airbus E-Fan X, NASA’s SUGAR Volt, and Rolls-Royce hybrid-electric architectures (e.g., turboelectric and parallel hybrid) explore megawatt-class propulsion systems, distributed electric propulsion, and the use of superconducting materials to improve power density and aerodynamic performance [7].

The transition toward MEA and AEA has created a pressing need to increase the operating voltage in onboard power systems [10]. As electric propulsion systems scale up to MW levels, a higher voltage is required to avoid extremely high current levels that would otherwise result in excessive resistive losses and significantly heavier cabling. For instance, while early MEA platforms operated at 270 V DC, current designs for future narrow-body AEA platforms, anticipate distribution voltages in the range of 1.5 to 3 kV DC to support power demands exceeding 20 MW [11,12]. This shift allows for improved system efficiency, lighter electrical infrastructure, and better thermal performance under high-load conditions. High-voltage architectures are especially critical in distributed propulsion configurations, where multiple electric machines must be supplied efficiently from a centralized or modular energy source. Furthermore, studies show that DC architectures at elevated voltages reduce the complexity of frequency management and improve compatibility with wide-bandgap power electronics [3,11].

In HVDC distribution systems, the design of insulation and protection becomes increasingly sensitive to environmental factors, especially atmospheric pressure [13]. In this context, Paschen’s Law provides a fundamental theoretical basis for how the breakdown voltage of a gas (such as air) depends on the product of pressure (*p*) and gap distance (*d*) between electrodes [14]. The law is commonly expressed as follows:

(1) $$V_{\mathrm{breakdown}}=\frac{B\cdot p\cdot d}{\ln\left(A\cdot p\cdot d\right)-\ln\left[\ln\left(1+\frac{1}{\gamma_{\mathrm{se}}}\right)\right]},$$

where $V_{\mathrm{breakdown}}$ is the breakdown voltage, and *A* and *B* are gas-dependent constants. In the case of air, A≈112.5 (Pa·m${)}^{-1}$ and B≈2737 V/Pa·m. The first Townsend ionization coefficient, which provides an idea of the number of ions available per unit length, is defined in terms of *A* and *B* as $\alpha =A\;p\;e^{-Bp/E}$, with *E* as the electric field. $\gamma_{\mathrm{se}}$ is the secondary electron emission coefficient and represents the number of particles available after an initial collision. According to Paschen’s curve, at low pressures, such as those encountered at typical cruising altitudes (e.g., ∼10 kPa), the breakdown voltage decreases sharply before rising again. This creates a critical vulnerability zone in high-altitude aircraft systems, where air becomes more prone to electrical breakdown at relatively moderate voltages. This effect has been experimentally observed in partial discharge testing of aviation cables, which showed increased instability and a higher risk of dielectric failure under low-pressure conditions [15]. Consequently, high-voltage system designers must account for Paschen’s law when evaluating insulation performance, spacing requirements, and overall system reliability in electric aircraft.

The appearance of an ionization phenomenon, such as electric arcs, could cause severe damage, as stated in the civil normative DO-160 [16]. Arc faults are a prevalent issue in aircraft wiring systems and represent a critical risk to operational safety [17].

Series arcs are difficult to detect because the associated impedance does not produce a significant drop in current, making standard overcurrent protection methods ineffective. As a result, series arcs may persist undetected for extended durations, potentially leading to progressive degradation of the wiring insulation and an increased risk of fire [18]. In addition, system noise from inverters and environmental variations can cause false positives in arc fault detection, challenging the stability and reliability of monitoring systems. The lack of standardized thresholds and the variability of field conditions further complicate accurate arc detection and limit the practical deployment of protection strategies [19].

A wide variety of detection strategies have been proposed in recent literature, leveraging different sensor types, frequency analysis ranges, and hardware implementations. Robles et al. [20] propose a highly efficient, low-weight, high-frequency current transformer (HFCT) specifically designed for more electric aircraft (MEA) environments. The sensor uses a ferrite toroidal core with optimized turns (e.g., N = 3) and achieves a sensitivity greater than 13 V/A within a bandwidth ranging from hundreds of kHz to tens of MHz. The sensor is passive, compact, and lightweight, making it ideal for onboard aviation systems. Compared to both commercial HFCTs and planar spiral coils, the proposed ferrite design demonstrates superior performance in detecting high-frequency pulses induced by serial arc faults. In addition to this approach, the same authors presented a modeling and experimental framework for air-core inductive sensors aimed at detecting arc-induced high-frequency components in HVDC aircraft wiring. Through finite element simulations and laboratory measurements, they demonstrated that a two-turn rectangular coil provides a favourable balance between sensitivity, integration complexity, and frequency response. Their results confirm that arc events generate significant energy in the range of hundreds of kHz and tens of MHz, which can be reliably captured by lightweight, non-intrusive magnetic sensors [21].

Similarly to aircraft electric systems, in photovoltaic (PV) systems, the adoption of high-voltage strings (often 600–1500 V DC) exacerbates the risk of ionization phenomena, particularly due to long cable runs, exposed connectors, and material degradation [19]. Both aircraft and PV electric systems present similar problems and propose different approaches. Sensor-based methods in high-impedance photovoltaic systems include the use of resonant RLC filters designed to passively amplify arc-induced frequency components (5 kHz), as described by Park et al. [22]. This approach provides a simple yet effective way to enhance arc detectability without complex signal injection or digital processing.

High-resolution digital techniques also play a significant role. Artale et al. [23] use the Chirp-Z transform (CZT) to perform spectral analysis in the 0–500 Hz range, capturing variations in low-order harmonics. This low-bandwidth approach allows reliable arc detection using short observation windows, which is computationally efficient. Meanwhile, Ahn et al. [19] apply a differential discrete wavelet transform (DWT) to analyze signals in the 60–200 kHz band. By introducing an adaptive thresholding mechanism, their method maintains high sensitivity across varying operating conditions and loads. It is implemented using a Rogowski coil and a DSP platform, allowing real-time detection in PV systems.

Xiong et al. [24] utilize passive shunt capacitors and Rogowski coils to measure arc-induced high-frequency components (1 kHz–1 MHz), leveraging FFT analysis and pulse polarity to both detect and localize arc faults in DC distribution systems. Their method is robust to system transients and provides directional information.

Reflectometry-based techniques, such as time-frequency domain reflectometry (TFDR), are employed by Bang et al. [25] for fault classification in multicore cables. These methods are powerful for diagnosing structural faults and involve wideband signal injection and machine learning-based image classification, but they are less suitable for real-time arc detection due to their complexity and hardware requirements. Sensor designs optimized for HF pulse detection (e.g., [20,22,24]) are particularly promising for DC arc fault detection in environments where weight, size, and electromagnetic interference are critical considerations.

In terms of instrumentation, most existing approaches for series arc detection rely on commercial off-the-shelf sensors with bandwidths below the hundreds of kilohertz range, aimed at identifying slow variations in DC current during arcing. As a result, detection times are typically in the order of hundreds of milliseconds, which is incompatible with the stringent safety requirements of aircraft systems. Consequently, prior research based on such sensors has focused on signal processing techniques to reduce detection times by identifying distinctive arc-related features, rather than on the development of new sensor technologies.

In contrast, our approach centers on the design of a novel sensor that detects the high-frequency (HF) pulses generated by arcs and propagated through the aircraft wiring. These pulses are characterized by durations on the order of hundreds of nanoseconds, enabling significantly faster arc detection and offering a path toward more responsive and reliable fault protection in aerospace electrical systems.

Capacitive sensors are non-intrusive devices that detect variations in electric fields through capacitive coupling between a high-voltage conductor and an external electrode. A simple yet effective capacitive sensor is proposed that is constructed by wrapping a copper tape around an insulated DC cable, forming a cylindrical electrode placed in close proximity to the energized conductor. This configuration creates a capacitive voltage divider, where the cable core and the copper tape act as the two electrodes of a capacitor, and the cable insulation serves as the dielectric medium. Any rapid voltage variation (such as those produced by series arc events) induces a displacement current across this capacitive interface. This makes the sensor inherently sensitive to high-frequency components and sharp transients, which are typical in arc fault signatures. As detailed by Nentzl and Plath [26], capacitive sensors can be implemented externally using metallic foils wrapped around the cable without shielding or internally by integrating the sensor into cable accessories such as joints or terminations in shielded cables. For example, the stress cone electrode or an internal foil insulated from the cable screen can act as a capacitive sensing element. These internal designs inherently provide shielding from stray coupling effects, improving measurement accuracy. Internal capacitive sensors typically operate in the MHz range to optimize the signal-to-noise ratio and penetration through the cable’s outer semiconducting layer.

Capacitive sensors have been widely used for partial discharge (PD) detection, with typical effective frequency ranges from 10 kHz up to 100 MHz [27]. While their application to series arc fault detection is not known to the authors, their physical behavior suggests strong potential in this domain, especially considering that series arcs emit broadband high-frequency noise in the 100 kHz to tens of MHz range [20]. According to Son et al. [28], capacitive coupling is one of the standard methods for electrical PD detection, alongside inductive and galvanic techniques. Capacitive sensors capture the high-frequency voltage pulses generated by PDs, with typical sensors being coupling capacitors or broadband capacitive couplers. For instance, Damião et al. [29] implemented a capacitive coupling technique to monitor partial discharges online in power transformers by leveraging the tap of condenser bushings. Their system operated effectively in a wide frequency range, up to 200 MHz. This demonstrates the practical viability of non-intrusive capacitive sensors for capturing fast electrical transients.

Capacitive sensors offer several advantages for non-intrusive electrical monitoring. They are inherently lightweight, compact, and cost-effective, making them well-suited for applications with strict constraints on space and mass, such as aerospace or photovoltaic systems. Their non-contact nature ensures galvanic isolation from high-voltage conductors, improving safety and simplifying installation without the need to break or modify existing wiring. Additionally, capacitive sensors are particularly sensitive to high-frequency transients, enabling the detection of phenomena such as partial discharges, and arc faults that produce rapid voltage changes [30].

This manuscript presents an adaptable model of a capacitive sensor designed for lightweight and non-intrusive integration in aircraft electric systems. It has been constructed by applying a copper tape over an insulated aerospace-grade wire, forming a cylindrical capacitor that couples to the electric field generated during arc events. Its expected capacitance and interaction with the cable and connections are derived analytically in Section 2 and validated through both simulation and experimental measurements, showing a strong correlation. The time-domain and frequency-domain responses of the sensor demonstrate its ability to detect high-frequency components associated with DC series arcs.

## 2. Capacitive Sensor Model

**Figure 1:**
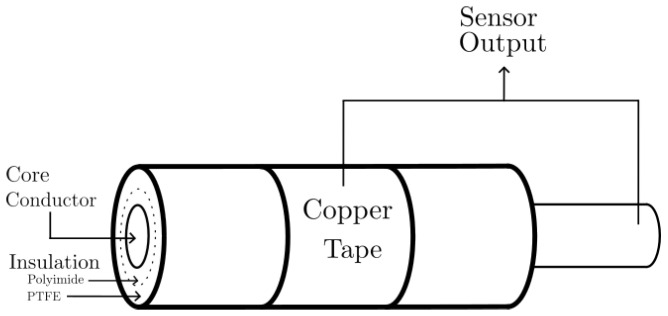
Capacitive sensor cross-section.

The capacitive sensor is constructed by placing a 2.5 cm adhesive strip of copper tape wrapped around an insulated and unshielded wire of 67 cm in length. The wire, model EN2267-010A220S (Prysmian Group, Milan, Italy), used in aeronautical electrical power systems, is effectively divided into two sections of 51 cm and 16 cm on both sides of the sensor. The copper tape serves as one plate of the capacitor, while the conductor inside the wire acts as the second plate (Figure 1).

**Figure 2:**
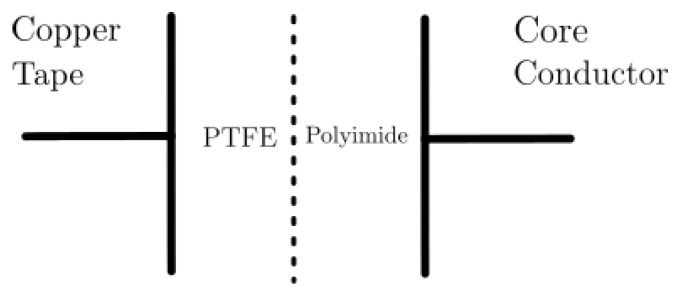
Equivalent capacitive sensor.

The wire has two insulating layers consisting of equal parts of reinforced polyimide and PTFE (Polytetrafluoroethylene) [31] (Figure 2). The overall relative permittivity, $\varepsilon_{r}$, of the dielectric can be calculated assuming that the total capacitance behaves as a combination of two capacitors in series, because of the layered configuration of the materials across the electric field direction. 

Equation (2) describes each capacitance value, $C_{1}$ and $C_{2}$, taking into account their respective dielectric constants and dimensional configurations and *C*, the total capacitance with an equivalent permittivity, $\epsilon_{r}$. These expressions are derived from the theoretical capacitance for a cylindrical capacitance [32] configuration analogous to a coaxial structure.

(2) $$C_{1}=l\frac{2\pi \varepsilon_{0}\varepsilon_{r1}}{\ln(R_{m}/r)},\;\;C_{2}=l\frac{2\pi \varepsilon_{0}\varepsilon_{r2}}{\ln(R/R_{m})},\;\;C=l\frac{2\pi \varepsilon_{0}\varepsilon_{r}}{\ln(R/r)},$$

where *l* is the width of the copper tape, *R* is the external radius, measured as the distance from the center of the conductor to the copper tape, which is 3.75 mm, and *r* is the internal radius, measured as the distance from the center of the conductor to the inner dielectric layer, which is 2.75 mm. $R_{m}$ represents the intermediate radius that separates the two dielectric regions within the cylindrical capacitance (Equation (3)). It corresponds to the midpoint between the inner conductor radius *r* and the external radius *R*.

(3) $$R_{m}=\frac{R+r}{2}.$$

In these equations, we assume that the total capacitance is calculated between the sensing plate and the section of wire located within its area, even though the second electrode is the entire length of the wire. This assumption is based on the idea that the electric field is primarily concentrated within this localized geometry, and that fringing effects are negligible. This is a valid approximation given that the length of the coaxial capacitance (25 mm) is significantly greater than its width (1 mm), ensuring a quasi-uniform field distribution.

The equivalent permittivity can be derived considering that $C^{-1}=C_{1}^{-1}+C_{2}^{-1}$ and solving for $\epsilon_{r}$ provides the value in Equation (4).

(4) $$\varepsilon_{r}=\frac{\ln(R/r)}{\frac{1}{\varepsilon_{r1}}\ln\left(R_{m}/r\right)+\frac{1}{\varepsilon_{r2}}\ln\left(R/R_{m}\right)}.$$

Since the permittivities of the Polyimide and PTFE are $\varepsilon_{r1}=3.4$ and $\varepsilon_{r2}=2.1$, respectively [31], the effective relative permittivity $\varepsilon_{r}$ is calculated to be 2.65.

Applying the analytical expression, the resulting theoretical capacitance of the capacitive sensor yields 11.88 pF.

The capacitive coupling sensor can be seen as a low impedance path to ground for the impulsive current signals created by a series arc in the wire under test. To test the sensor response to this type of signal, the section of wire is connected to a signal generator with a bandwidth of 250 MHz. A step voltage with a rise time of 2.5 ns is applied to the section of wire exciting frequencies approximately up to $0.35/2.5\times 10^{-9}=140$ MHz. The step response in the time domain provides information about the sensor itself as well as the setup connected to the signal generator.

A theoretical analysis is carried out on the parameters that affect the step response signal, enabling the estimation of each component. The inductance of the connecting wires and the sensor capacitance are determined based on their geometric parameters.

**Figure 3:**
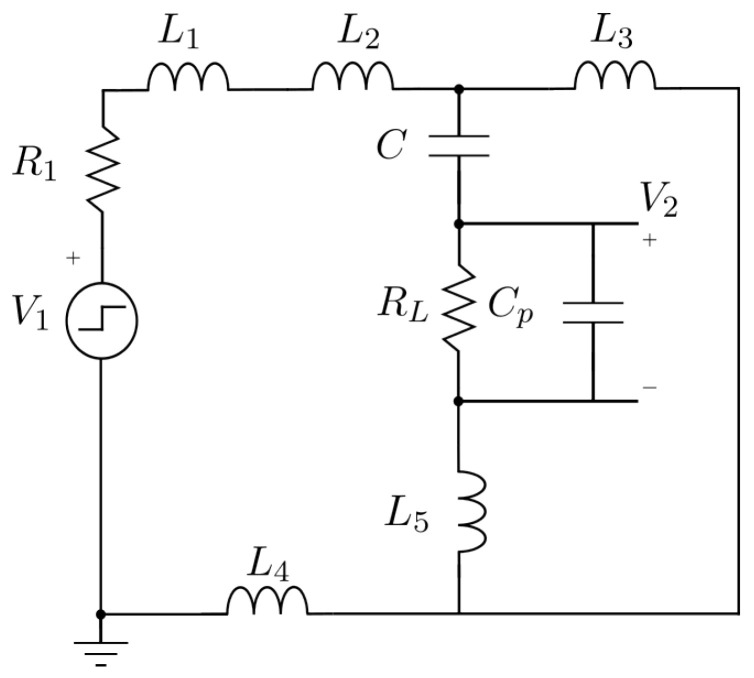
Theoretical model schematic of the capacitive sensor.

The equivalent circuit is shown in Figure 3, where $V_{1}$ models the step generated by the function generator, $R_{1}$ represents its 50 Ω output impedance, $L_{2}$ and $L_{3}$ are the two sections of the wire under test on both sides of the coupling sensor, and $L_{1}$, $L_{4}$, and $L_{5}$ correspond to the inductances associated with the connecting wires. $R_{L}$ is the 50 Ω input impedance of the oscilloscope. $C_{p}$ represents the capacitance of the coaxial cable connecting the capacitor to the oscilloscope, together with the oscilloscope’s input capacitance. $V_{2}$ is the observed output signal at the oscilloscope. Finally, *C* denotes the capacitance of the capacitive sensor.

The inductance associated with the cable under test and the connecting wires is based on the total self-inductance of a long conductor with current uniformly distributed over its cross-section [33] provided by Equation (5):

(5) $$L=\frac{\mu_{0}}{2\pi }\ell_{i}\left[\ln\left(\frac{2\ell_{i}}{r_{i}}\right)-k\right],$$

where *L* is the inductance, $\ell_{i}$ is the length of the wire, and *r* is the radius of the core *i* conductor. The constant *k* varies with both the frequency and the geometry of the conductor. Its value typically ranges from 0.75 for low-frequency or DC conditions, up to 1 at high frequencies, where the internal magnetic field within the conductor becomes negligible [34]. $R_{2}$ in Equation (6) is the total resistance of the wire under test where the capacitive sensor is integrated, and its value increases with frequency due to the skin effect [35].

(6) $$R_{2}=\frac{\ell_{i}}{2\pi r_{i}\sigma_{\mathrm{Cu}}\delta_{\mathrm{Cu}}},$$

with

(7) $$\delta_{\mathrm{Cu}}=\frac{1}{\sqrt{\pi f\mu_{\mathrm{Cu}}\sigma_{\mathrm{Cu}}}},\;\delta_{\mathrm{Cu}}=\frac{0.066}{\sqrt{f}},$$

where $\delta_{\mathrm{Cu}}$ is the skin depth, $\mu_{\mathrm{Cu}}≃\mu_{0}$ is the permeability of copper, $\sigma_{\mathrm{Cu}}=5.8\times 10^{7}\;S/m$ is the conductivity, and *f* is the frequency. For a frequency of 140 MHz, which is the maximum expected in this study, and a conductor with a radius of 2.75 mm, $R_{2}$ is equal to 120 mΩ and $\delta_{\mathrm{Cu}}$ = 5.57 μm. These results have two consequences: first, $R_{L}\gg R_{2}$, so the voltage drop across the maximum lumped cable resistance $R_{2}$ can be assumed to be negligible. Second, the constant *k* in (5) is set to 1 because the skin depth $\delta_{\mathrm{Cu}}$ of copper at the maximum frequencies of interest is very small. Under these high-frequency conditions, the conductor effectively behaves as if all the current flows on its surface [34].

**Figure 4:**
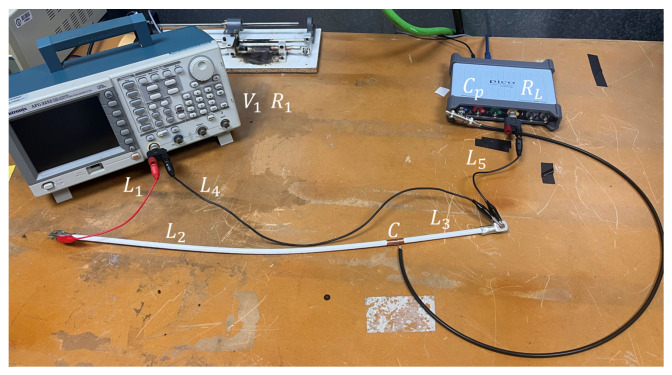
Experimental setup for step response measurements in the laboratory.

The cables used in the experimental setup (Figure 4) were carefully measured, as their lengths directly affect the inductance values assigned to each segment in the theoretical model. $L_{1}$ corresponds to the red cable from the signal generator with a length of 25 cm. $L_{2}$ corresponds to the left section of the white cable under test, with a length of 51 cm, and $L_{3}$ represents the right segment of the same white cable, measuring 16 cm. This cable is the one shown in Figure 1, on which the copper tape was applied to construct the capacitive sensor, *C*. $L_{4}$ corresponds to the cable that connects the ground of the function generator to the white coaxial cable, with a length of 61 cm. Notice that this cable length is similar to the cable under test. The reason is that we do not want to bend the cable to reduce the connecting wires and keep it as straight as possible. $L_{5}$ is the cable connecting the oscilloscope ground to the white coaxial cable, measuring 25 cm. Therefore, $\ell_{1}=25$ cm, $\ell_{2}=51$ cm, $\ell_{3}=16$ cm, $\ell_{4}=61$ cm, and $\ell_{5}=25$ cm; furthermore, $r_{1}=r_{2}=2.8$ mm and $r_{3}=r_{4}=1$ mm.

From the above expression, the following inductance values were obtained: $L_{1}=1.18\;\mu H$, $L_{2}=0.98\;\mu H$, $L_{3}=0.75\;\mu H$, $L_{4}=1.36\;\mu H$, and $L_{5}=1.18\;\mu H$.

Finally, $C_{p}$ is the capacitance of an RG58 coaxial cable that is 26 cm in length with a capacitance of 100 pF per meter in parallel with the input capacitance of the oscilloscope, 14 pF, and is estimated to be 40 pF.

Table 1 lists the calculated electrical parameters for the inductances and capacitance in the circuit, obtained from the analytical expressions presented earlier. Table 2 provides a summary of the geometric dimensions of the circuit elements, which were used to estimate the inductance and capacitance values based on the physical configuration of the experimental setup.

Once the circuit is modeled, a simulation is carried out in Simulink to observe the system’s response to a step input of 5 V. This simulation provides a theoretical reference to compare with the experimental data, allowing the validation of the model. The labels of each component correspond to the theoretical schematic shown in Figure 3.

**Figure 5:**
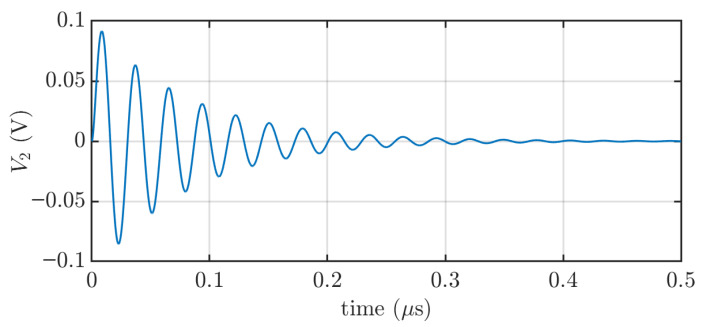
Step response of the capacitive sensor in Simulink.

As expected, considering the combination of inductances, capacitances, and resistances, the simulated output voltage $V_{2}$ (Figure 5) exhibits a damped oscillatory response with a damped oscillation frequency of 35 MHz.

## 3. Model Validation

**Figure 6:**
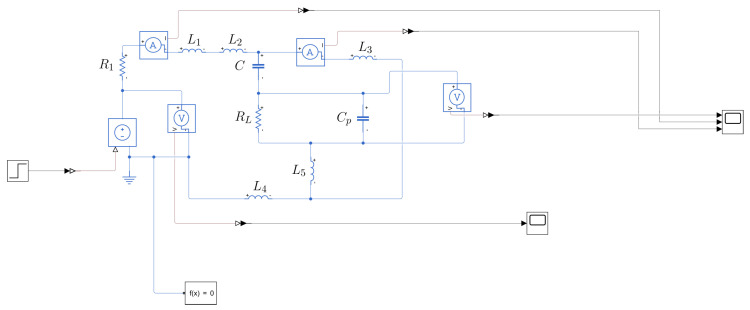
Theoretical model schematic of the capacitive sensor in Simulink.

After completing the theoretical modeling, experimental measurements were conducted to validate the proposed model. The setup of the circuit, as assembled in the laboratory, is shown in Figure 4. The labels of each component correspond to the theoretical schematic shown in Figure 3 and Figure 6.

The step signal with a rise time of 2.5 ns used to excite the circuit was generated using a function generator with an output impedance of 50 Ω, matching the value used for $R_{1}$ in the theoretical and simulation model.

These characteristics ensured that the experimental input closely mirrored the theoretical step input, allowing for a direct comparison between the simulated and measured responses.

The oscilloscope, with an input impedance of 50 Ω ($R_{L}$), is represented by $V_{2}$. The sampling rate is 250 MHz, the time window is set to 1 ms (corresponding to 100 μs/div), and the resolution is 12 bits. Setting the time window so large for such a short time response in the range of 500 ns is to have a fine resolution in frequency when calculating the frequency response.

**Figure 7:**
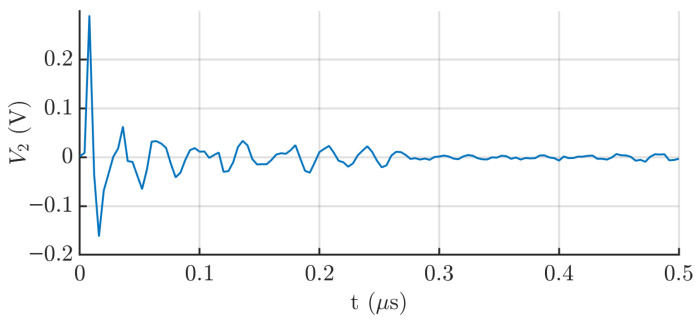
Step response of the capacitive sensor.

**Figure 8:**
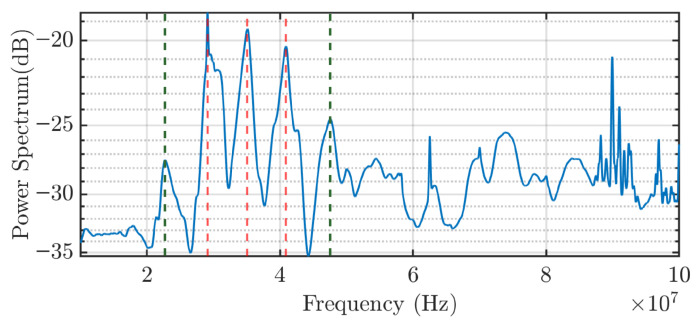
Frequency response of the experimental response. The red and green lines indicate frequency peaks corresponding to reflections in the transmission line.

The experimental response of the capacitive sensor, as observed on the oscilloscope, is shown in Figure 7. The output also exhibits a damped oscillatory signal, with a frequency around 35 MHz. This value is consistent with the oscillation frequency obtained from the Simulink simulation, which supports the validity of the theoretical circuit model, and especially, the value of the coupling capacitance. As shown in Figure 8, which presents the signal spectrum, a clearly defined peak appears around 35 MHz. This value corresponds to the frequency with the highest peak in the power spectrum (dB), indicating that it is the dominant frequency component of the signal detected by the sensor.

As revealed in Figure 8, the frequency response does not exhibit a single peak. The central peak corresponds to the response to the step as in the modeled circuit, while additional sidebands appear at frequency offsets of approximately 6 MHz (red lines) and 12 MHz (green lines). These sidebands indicate the presence of reflections in the transmission line. This behavior is expected due to cable splices and mismatches in impedance between different cable segments.

These additional sidebands explain why the theoretical signal differs from the experimental one in terms of attenuation. Although both are similar in structure, the sidebands cover a wider frequency range, which translates in the time domain into interference over the initial damped oscillation. As a result, the signal does not appear as a clean damped sinusoid, but rather as a dominant oscillatory component modified by interfering signals. Consequently, certain portions of the waveform are attenuated, even though the central frequency of the oscillation remains present throughout. Despite these deviations, the theoretical values previously calculated are considered valid, as the core dynamic behavior of the system is preserved.

These results validate the model of the cable segment and the value of the coupled capacitance, opening the possibility of integrating a sensor in a more complicated electric circuitry in real applications. The aim in the next section is to determine a configuration of the sensor that increases the sensitivity to high-frequency pulses generated by series arcs.

## 4. Parametric Analysis

To study the behavior of the circuit modeling the capacitive sensor and measuring setup (Figure 6) to changes in the length of wires and geometry of the coupling, five parametric simulations were conducted taking the basic configuration explained in Section 2:

1. Wire lengths at inductances of $L_{2}$ and $L_{3}$ were increased ten-fold.
2. The coupling capacitance, *C*, was multiplied by a factor of ten.
3. Wire length and coupling capacitance, including all electrical parameters, were increased tenfold.
4. Wire length was increased and connecting wire lengths were decreased; all inductances were modified by a factor of ten.
5. Wire length and coupling capacitance we increased simultaneously, along with a reduction in connecting wires.

All changes were applied systematically to assess their individual and combined effects on resonant frequency, peak amplitude, and transient settling time.

In the baseline configuration (shown in Table 1) and setting a step signal of 5 V, the circuit resonates at 35 MHz exhibit a settling time of 0.4 μs, reaching a peak amplitude of 91.5 mV. When $L_{2}$ and $L_{3}$ are increased by a factor of ten, the resonance nearly halves to 19.1 MHz, the peak rises to 121.62 mV, and the settling time grows to 1.4 μs, demonstrating the classic lowering of resonant frequency and slower response with higher inductance. Increasing only the sensor capacitance by ten-fold shifts the resonance further down to 10.98 MHz, boosts the peak to 235.09 mV, and yields a settling time of 0.5 μs, indicating that larger coupling capacitance amplifies voltage swings but modestly delays stabilization. In case three, when both the length of the wire and the coupling capacitance are multiplied by ten, the circuit resonates at just 6.06 MHz, with a peak of 334.51 mV and settling time 1.6 μs, confirming the additive effects on frequency reduction and transient elongation. In the fourth scenario, when $L_{2}$ and $L_{3}$ are increased by a factor of ten, the connecting wires are reduced and $C_{p}$ is reduced from 40 pF to 17 pF. The resonance rises to 22.25 MHz and the peak to 158.92 mV, but the settling time increases to 1 μs, illustrating how reduced parasitic capacitance and measurement-cable inductance can speed oscillation yet prolong the transient envelope. Finally, by additionally multiplying the coupling capacitance of the base configuration by 10 in case five, the frequency drops to 6.85 MHz, the peak surges to 421.13 mV, and the settling time extends to 0.8 μs, underscoring that a large coupling dominates both amplitude enhancement and settling delay even when measurement inductances are minimized. Overall, these results confirm the inverse relationship of resonant frequency with inductances and capacitances, the direct dependence of voltage amplitude on the coupling capacitance, and the tendency of settling time to increase with stored energy in reactive components.

Table 3 summarizes the parameter variations and corresponding resonance frequency, peak amplitude, and settling time observed across the five simulation scenarios.

In conclusion, to achieve optimal performance of the coupling capacitance for acquiring high-frequency current pulses generated by series arcs, the coupling should be maximized by either increasing the length of the capacitor or replacing the two insulating layers with a dielectric material of higher permittivity. Additionally, the connecting wires should be kept as short as possible, and the input capacitance of the acquisition system should be minimized to reduce signal attenuation and preserve high-frequency content.

## 5. Experimental Measurements with Series Arcs

The testing signals are actual series arcs created in the High-voltage Research and Test Laboratory (LINEALT) at the University Carlos III of Madrid using a 2 kW variable resistive load connected to a 270 V direct current voltage source set to two different current levels of 3 and 5 A. The voltage setting is a standardized value in aeronautical electric systems [16], and the current setting is from typical loads.

Arcs are generated using two different methods. The first involves separating two electrodes while a current of 5 A is flowing. The aim of this experiment is two-fold: firstly, to demonstrate the feasibility of the capacitive sensor to detect arcs and, secondly, to show how the power of the signal captured with the capacitive sensor increases when the arc is active. To this end, the power of a signal is computed in no-arc and arc conditions and compared in a table.

The second method uses a section of cable loosely connected to the main conductor and mounted on a shaker to induce arcing through mechanical vibrations. The setup changes substituting the longitudinally moving electrodes with the shaker. In this case, the objective is as follows:

- The performance of the capacitive sensor is validated in another setup with different components and wiring configurations.
- The DC current is reduced to 3 A; therefore, the sensor is tested based on variations in the arc current.
- An inductive sensor, already tested in series arc measurements, is included to compare the results obtained with the capacitive sensor.
- Most importantly, the capacitive sensor is validated with an arcing event with multiple events that differ significantly from the arcs generated in the first setup.

### 5.1. Setup with Arc Generator

In the first experiment, the arc is generated using a controlled switch that gradually increases the distance between two electrodes. This approach interrupts the current flow while still allowing the passing of electrons in the form of plasma. This type of arc generator was previously presented in [30]. However, in the present case, the electrodes consist of cylindrical copper pipes with a thickness of 6 mm, chosen because copper is the most common conductor material used in aeronautical systems.

The interruption mechanism relies on a stepper motor connected to a lead screw, which moves one of the electrodes longitudinally. The motor is controlled by a motor driver, which is in turn managed by a microcontroller. Parameters such as the electrode separation speed and the final distance between them can be configured through an Android application over Wi-Fi. In all tests conducted, the electrodes were separated by at least 5 cm to ensure arc extinction. The complete separation process took 800 ms, which is the maximum speed achievable with the current driver setup.

Several electrode separation speeds were evaluated, and the results showed that the sensor’s signal remained unaffected by the variation in electrode separation rate. This confirmed the sensor’s ability to detect both short-duration flashover and sustained arcs equally well.

**Figure 9:**
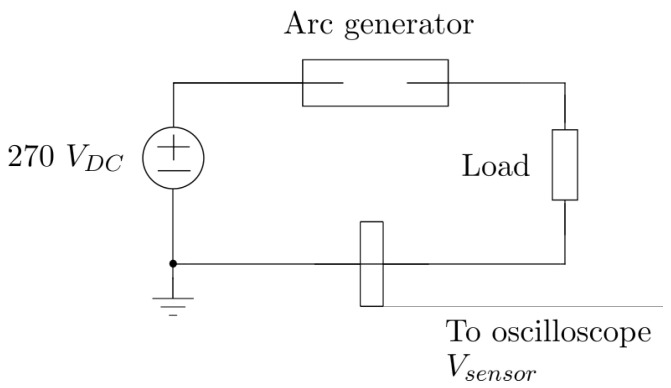
Experimental setup for arc measurements as a schematic.

During the tests, the capacitive sensor under evaluation measured the leakage current to ground due to electric field variations associated with the high-frequency (HF) voltage transients present in the power cable that connects the source, the controlled switch, and the load (Figure 9).

### 5.2. Setup with Vibrations

**Figure 10:**
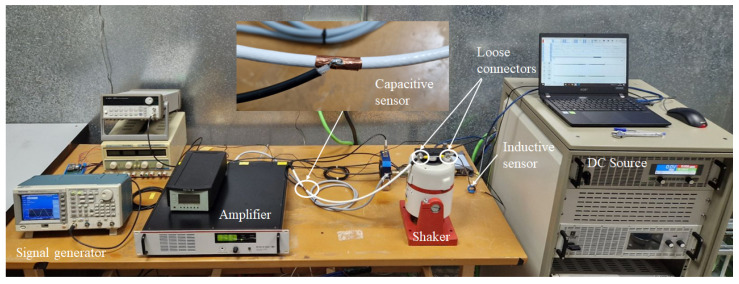
Experimental setup for arc measurements using a shaker to create arcs through the vibration of loose cable connectors.

This is a more advanced setup (see Figure 10) that includes a shaker to induce arcing through the vibration of loose connections, a failure mode more representative of those that may occur in aerospace systems. The loose connections are created using a section of cable connected to two terminals with loosely tightened nuts. This assembly is then mounted on the shaker to simulate vibration-induced arcing.

In this setup, the DC current is limited to 3 A, as temperatures at the loose connections exceeded 300 °C in less than 30 s during testing, causing the insulation of the cable section to melt. It is important to note that, during vibration, the contact area between the wire terminal and the screw–nut assembly is much smaller than in a properly tightened connection. This reduced contact area increases the current density, leading to significant heat generation. This highlights the severity and potential hazard of such events in aerospace environments.

The amplitude and frequency of the vibration are controlled using a signal generator and an amplifier connected to the shaker. For the following experiments, the vibration frequency was set to 60 Hz, although both higher and lower frequencies have also been tested. Arc ignition depends on the relative position of the terminal with respect to the screw and nut: when they are in contact, current flows normally without arcing; however, when they separate, an arc can strike from the screw thread to the wire terminal. The presence of the thread introduces a degree of randomness in the contact dynamics, resulting in a desirable lack of correlation between arc events and the vibration frequency.

To compare the performance of the capacitive sensor, an inductive sensor designed by the authors in [20] was also included. This sensor was placed around the main conductor and provided a signal proportional to the derivative of the current through it.

## 6. Discussion of the Sensor Performance

### 6.1. Results Obtained with the Arc Generator

**Figure 11:**
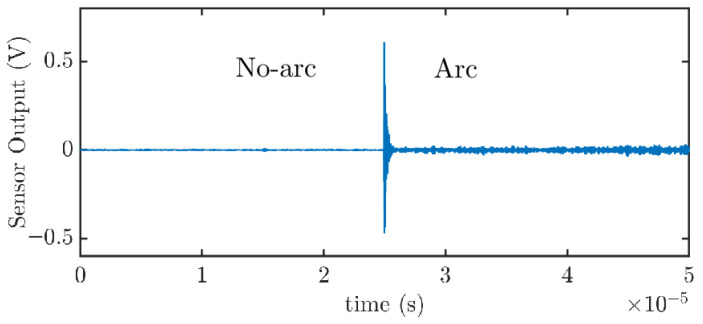
Excerpt of a signal taken with the proposed capacitive sensor to determine the power before and after the arc inception.

High-frequency (HF) current pulses generated by electric arcs are inherently stochastic, with durations of only a few microseconds and temporal separations that may span several hundred microseconds [20]. An increase in the power of the current signal captured by the capacitive sensor is indicative of heightened arc-related pulsing activity. To assess the sensor’s performance, the ratio of the signal power during arc events to that during non-arc conditions is computed. Signal power is determined in the time domain by calculating the mean of the squared signal samples, ensuring that all frequency components are accounted for. Notably, a significant rise in the HF content of the current, relative to the baseline noise level in the absence of arcing, is a key indicator for detecting arc initiation and monitoring its behavior. Therefore, the analysis of signal power focuses on two distinct time intervals: a segment before the arc ignition (no arc) and an equally long segment immediately after the arc event (arc). The power analysis will concentrate on the signal segment spanning an equal duration before and after the trigger point. In the waveform shown in Figure 11, the arc is clearly identified by a sharp transient in the sensor output, occurring at 25 μs. The no-arc portion precedes this event and exhibits minimal activity, representing the background noise level. The arc portion follows the transient and contains high-frequency components resulting from the arc-induced current impulses [20]. These signals span a wide frequency band, determined by the electrical characteristics of the system, specifically, the load response, cable geometry, and the position of the capacitive sensor relative to junctions and connectors. These factors significantly influence how the arc energy is distributed across the frequency spectrum [20], as demonstrated in Section 3.

For instance, if the total acquisition time is 50 μs and the trigger occurs at 25 μs, the analyzed portion of the signal will cover the 50 μs, comprising 25 μs preceding and 25 μs following the trigger (see Figure 11).

In Table 4, the columns labeled “no-arc” represent the signal power during the first 25 μs, corresponding to the high-frequency components primarily caused by conducted noise from the power source, experimental setup, and transmission line. In contrast, the columns labeled “arc” show the signal power during the subsequent 25 μs interval, when the arc is present and active.

The results reveal a substantial increase in signal power when an arc is present. Specifically, the average power rises from $5.54\times 10^{-6}\;V^{2}$ under no-arc conditions to $5.017\times 10^{-4}\;V^{2}$ during arc activity, representing nearly a two-order-of-magnitude increase. This indicates that arc events inject a significantly higher amount of energy into the system. Furthermore, the standard deviation also increases considerably, from $8.80\times 10^{-6}\;V^{2}$ to $7.593\times 10^{-3}\;V^{2}$, highlighting the unstable and highly dynamic nature of arc-related signals.

**Figure 12:**
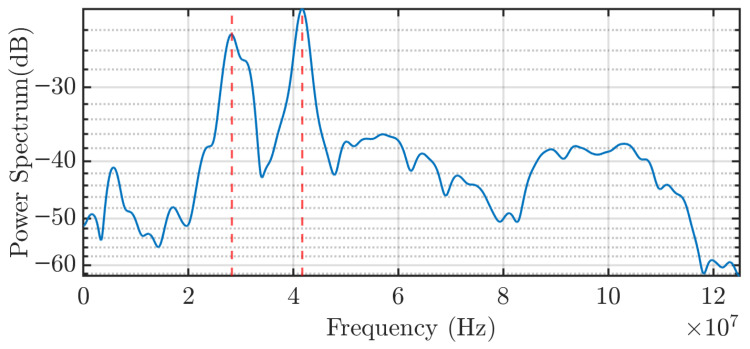
Spectrum of the capacitive sensor’s arc response. Red lines in the figure highlight two dominant peaks in the spectrum, centered around 28 MHz and 41.7 MHz.

**Figure 13:**
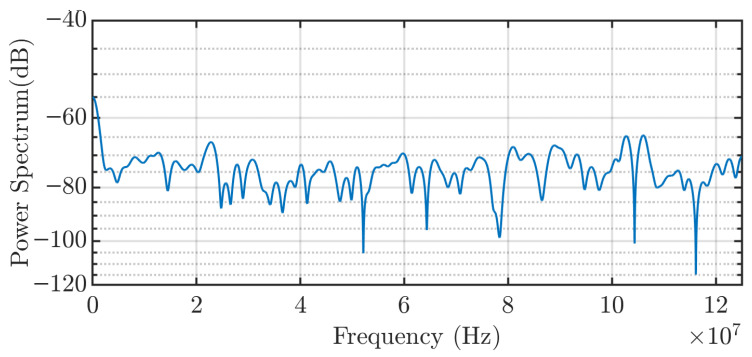
Spectrum of the capacitive sensor’s no-arc response.

Figure 12 displays the segment where the arc is present; the spectrum reveals two dominant peaks centered around 28 MHz and 41.7 MHz, while Figure 13 corresponds to the portion of the signal without arc activity. This signal corresponds to the HF components of a current flowing through the electrodes.

The shift in the frequencies observed in the model of the cable under test is due to the addition of three extra wire segments: from the arc generator to the load, from the load to the DC source, and from the DC source to the cable. These additional sections, along with the load, which can be represented by an additional RLC equivalent circuit, constitute a modified configuration that differs from the original characterization setup. As a result, the resonant frequencies present in the arc signals deviate from those initially identified.

### 6.2. Results Obtained with Vibrations

**Figure 14:**
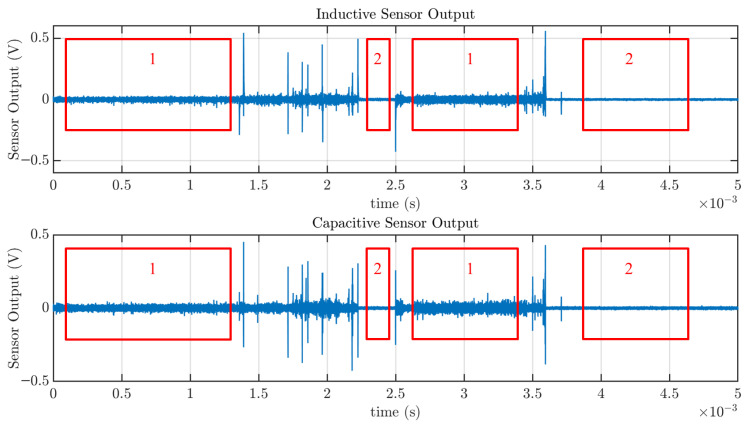
Example of arcing created with the vibration of loose connectors. The upper plot is captured with an inductive sensor, and the lower plot with the proposed capacitive sensor. Section 1 represents a portion of the signal with arcing, while Section 2 represents normal conduction. The spikes are the instances where connections and disconnections occur.

In the second experimental setup, the vibration causes repeated connections and disconnections of the wire, resulting in a large number of arcs in the form of sparking. The characteristics of the signals are similar to those obtained in the previous setup, but with a significant increase in activity (see Figure 14, where the upper plot corresponds to the signal captured with the inductive sensor and the lower plot to the capacitive sensor).

An increase in signal amplitude and power can be observed during arc activity, as indicated in the sections labeled ’1’, compared to sections labeled ’2’, which correspond to steady current flow when the terminals remain in contact with the screw threads. This behavior is consistent with the results obtained in the experiment involving longitudinal electrode separation.

To quantify the power increments, different segments of the signal containing arc activity and steady current flow were examined for both sensors. For the capacitive sensor, the measured power values in the most and least energetic sections were $14.3\times 10^{-5}\;V^{2}$ and $1.41\times 10^{-5}\;V^{2}$, respectively, yielding a ratio close to 10 between arc and non-arc conditions, which is significantly smaller than the ratio observed in the first experimental setup. The reduction is attributed to several factors: (i) the DC current is lower in this setup (3 A vs. 5 A); (ii) the electrode surface area in the first setup is larger, allowing for a wider plasma channel; (iii) the rapid connection and disconnection caused by vibration limits the formation of longer-lasting arcs. Altogether, these conditions result in lower energy being transferred during each arc event when compared to the first experiment.

**Figure 15:**
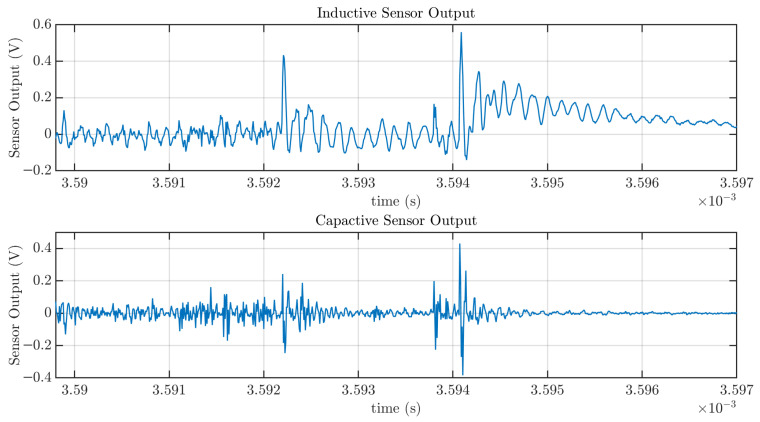
Detail of the signal shown in Figure 14 showing two current pulses acquired with the capacitive sensor and compared to the response of the inductive sensor.

For the inductive sensor, the corresponding power values were $24.52\times 10^{-5}\;V^{2}$ and $1.072\times 10^{-5}\;V^{2}$, respectively, providing a ratio of approximately 23. This larger variation, compared to the capacitive sensor, is attributed to the slower dynamic response of the inductive sensor, which results in longer-duration signals in response to current pulses, and consequently, greater integrated energy over time. This characteristic can be observed in the most remarkable feature of these signals, which is the spikes in both plots. They correspond to the moments of connection and disconnection, which involve sudden changes in current. These rapid transitions result in sharp current pulses detected by both the inductive and capacitive sensors. While the peak amplitudes are comparable for both sensors, differences become evident when zooming into the details of a single pulse, as shown in Figure 15. This difference is an inherent characteristic of capacitive sensors, which respond more rapidly to changes in current, whereas inductive sensors exhibit slower dynamics. This can be seen, for example, in the extended tail of the second large pulse in Figure 15, approximately from 3.594 ms to 3.597 ms.

The time window shown in Figure 14 spans only 5 ms. During the acquisition campaign, 131 signals were acquired (a total of 655 ms), each exhibiting similar behavior. This highlights the high activity of arcing in this experiment and reinforces the importance of prompt detection to prevent cable insulation from melting and potentially causing a fire.

## 7. Conclusions

The proposed sensor is constructed by wrapping a copper strip around an aerospace-grade insulated wire, forming a cylindrical capacitor that detects variations in the electric field during arc events and facilitates a low impedance path to ground for high-frequency currents. This design leverages the inherent sensitivity of capacitive sensors to high-frequency transients and rapid voltage changes, key characteristics of arc fault signatures.

The paper details a theoretical model of the sensor, validated through both simulation and experimental measurements in two different setups. The analytically calculated sensor capacitance of 11.88 pF showed strong correlation with experimental results. The sensor’s response in both time and frequency domains confirms its ability to detect high-frequency components associated with series DC arcs.

To achieve optimal performance of the coupling capacitance for acquiring high-frequency current pulses generated by series arcs, the coupling should be maximized by either increasing the length of the capacitor or replacing the two insulating layers with a dielectric material of higher permittivity in the section of the wire with the capacitor. Additionally, the connecting wires should be kept as short as possible, and the input capacitance of the acquisition system should be minimized to reduce signal attenuation and preserve high-frequency content.

For experimental testing with actual arcs, two controlled arc generators were used, capable of creating series arcs at 270 V DC and different current levels, which are typical conditions in aerospace systems. Both setups were designed to test the performance of the capacitive sensor. The first configuration creates arcs by separating two electrodes longitudinally. This generates a signal where it is easy to distinguish two sections: no arc and arc. Comparing the signal power in the arc and non-arc periods revealed a significant increase in high-frequency content during arc events, highlighting the sensor’s effectiveness in identifying arc activity. The experimental measurements exhibited some deviations in the frequency spectra compared to the baseline circuit used to validate the capacitive sensor. These differences are expected, as the test setup included additional cable sections, introducing new transmission line reflections due to cable splices and impedance mismatches. Moreover, the current limiting load in the circuit, which can be modeled as an RLC network, contributes to the altered dynamic response of the system. Despite these changes, the core behavior of the system was preserved: it consistently responded to high-frequency currents generated by series arcs. Consequently, both the theoretical model and the calculated coupling capacitance value remained valid.

The second setup was designed to generate arcs through the vibration of loose connections in a wiring system, simulating a more realistic and likely failure scenario. The objective of these measurements was to evaluate the performance of the capacitive sensor in comparison with a previously validated inductive sensor. In this case, the arc activity is more intense and exhibits different temporal dynamics, making it an ideal testbed for sensor comparison. The capacitive sensor reliably tracks the signals captured by the inductive sensor, demonstrating comparable sensitivity. However, differences become evident when examining individual current pulses in detail: the capacitive sensor exhibits a significantly faster response than the inductive sensor, a characteristic that could be used in future detection systems to reduce arc detection time.

In summary, the results of this work confirm the feasibility and performance of capacitive sensors for series arc detection in aerospace applications. These sensors offer significant advantages, such as their lightweight and non-intrusive design, low cost, and galvanic isolation from high-voltage conductors, which enhances safety and simplifies installation. The proposed approach demonstrates a promising step toward safer and more efficient arc detection systems for next-generation electric aircraft.

## Figures and Tables

**Table 1:** Calculated component values for the theoretical model schematic.

Label	Value	Unit
$L_{1}$	1.18	μH
$L_{2}$	0.98	μH
$L_{3}$	0.75	μH
$L_{4}$	1.36	μH
$L_{5}$	1.18	μH
C	11.88	pF

**Table 2:** Geometric dimensions of the circuit elements.

Label	Value	Unit
$l_{1}$	25	cm
$l_{2}$	51	cm
$l_{3}$	16	cm
$l_{4}$	61	cm
$l_{5}$	25	cm
R	3.75	mm
r = $r_{1}$ = $r_{2}$	2.75	mm
$r_{3}$ = $r_{4}$	1	mm

**Table 3:** Comparative results of circuit simulations with varying inductances and capacitances.

Sim.	$L_{2}$, $L_{3}$	$L_{1}$, $L_{4}$, $L_{5}$	*C*	$C_{p}$ (pF)	Res. Freq. (MHz)	Peak (mV)	Settling Time (μs)
0 (Baseline)	×1	×1	×1	40	35	91.5	0.4
1	×10	×1	×1	40	19.1	121.62	1.4
2	×1	×1	×10	40	10.98	235.09	0.5
3	×10	×1	×10	40	6.06	334.51	1.6
4	×10	÷10	×1	17	22.25	158.92	1
5	×10	÷10	×10	17	6.85	421.13	0.8

**Table 4:** Power measured between no arc and arc transitions.

No-Arc			Arc		
Avg.	Std.	Avg.	Avg.	Std.	Avg.
$V^{2}\times 10^{-6}$		dB	$V^{2}\times 10^{-5}$		dB
5.54	8.80	−52.56	50.17	759.30	−33.00

## Data Availability

The original contributions presented in this study are included in the article. Further inquiries can be directed to the corresponding author.

## Abbreviations

The following abbreviations are used in this manuscript:

AC	Alternating current
AEA	All-electric aircraft
CZT	Chirp-Z transform
DC	Direct current
DSP	Digital signal processor
DWT	Discrete wavelet transform
EASA	European Union Aviation Safety Agency
FFT	Fast Fourier transform
FM	Frequency modulation
HF	High frequency
HFCT	High-Frequency current transformer
HVDC	High-Voltage direct current
LINEALT	High-voltage Research and 346 Test Laboratory
MEA	More-electric aircraft
PD	Partial discharge
PTFE	Polytetrafluoroethylene
PV	Photovoltaic
RF	Radio frequency
TFDR	Time-frequency domain reflectometry
